# Choroidal vascular analysis in myopic eyes: evidence of foveal medium vessel layer thinning

**DOI:** 10.1186/s40942-017-0081-z

**Published:** 2017-05-26

**Authors:** Rayan A. Alshareef, Mohammed K. Khuthaila, Manideepak Januwada, Abhilash Goud, Daniela Ferrara, Jay Chhablani

**Affiliations:** 1 Department of Ophthalmology, McGill University, Montreal, QC Canada; 2 Department of Ophthalmology, King Faisal Specialist Hospital and Research Center, Riyadh, Saudi Arabia; 3 Srimati Kanuri Santhamma Retina Vitreous Centre, L. V. Prasad Eye Institute, L V Prasad Marg, Banjara Hills, Hyderabad, Telangana 500 034 India; 4 Department of Ophthalmology, New England Eye Center, Tufts Medical Center, Boston, MA USA

**Keywords:** Myopia, Choroid, Optical coherence tomography, Choroidal vessels

## Abstract

**Purpose:**

To analyse morphologic features of the choroid in Non-pathological myopic eyes using spectral-domain (SD) optical coherence tomography (OCT).

**Methods:**

Retrospective analysis of enhanced depth SD-OCT images of Non-pathological myopic eyes in comparison with age-matched healthy controls was performed. Choroidal thickness (CT) and large choroidal vessel thickness (LCVT) were measured at the fovea, 750 µm nasally from fovea (N^750^) and 750 µm temporally (T^750^) from fovea. Medium choroidal vessel thickness (MCVT) was calculated by subtracting LCVT from CT. Choriocapillaris was encompassed by MCVT, given its reduced thickness. Linear regression analysis evaluated the relationship between age and axial with CT, LCVT and MCVT.

**Results:**

The study group comprised 42 eyes of 31 patients (mean age 46.13 ± 15.63; 15 females). Control group included 57 eyes of 34 patients (mean age of 42.3 ± 15.29; 24 females). Mean axial length in myopic eyes and control group was 26.57 ± 1.27 and 23.59 ± 0.99 mm respectively. Myopic eyes showed significant thinning of MCVT and CT at all locations (p < 0.0001) compared to controls, unlike LCVT (p > 0.05). With each decade, thinning of up to 37 µm in CT was noted along with thinning of LCVT (up to 22.6 µm) and MCVT (up to 25 µm). Each mm increase in axial length caused 38.2 µm thinning of choroid along with LCVT (<10 µm), however, MCVT showed more notable thinning (>30 µm).

**Conclusion:**

Significant thinning of MCVT was noted in non-pathological myopic eyes in comparison to healthy subjects. It appears that MCVT has stronger relationship with age and axial length.

## Background

Myopia is a prevalent cause of vision impairment, although the cause and exacerbating factors are not completely understood. Histological analysis has revealed choroidal thinning in myopia, reported to be between 11 and 13 µm of thickness lost for each decade of life, and 6–9 µm lost with each dioptre of myopia [1]. Moreover, progressive choroidal thinning has been found to be age-related in normal eyes as well [2]. Despite abundant epidemiological evidence, the root cause or determinant of choroidal thinning in myopic eyes remains poorly understood. Some histopathological studies have suggested that choroidal thinning in myopia may be associated with a loss of large blood vessels [3], while others have postulated that this may result from pronounced thinning of the choriocapillaris [4].

Enhanced depth imaging (EDI) spectral-domain optical coherence tomography (SD-OCT) is an acquisition technique that places OCT images near the zero-delay line, so the roll-off of sensitivity does not compromise image registration. It provides better documentation of deeper structures such as the choroid and choroidal-scleral interface, therefore contributing to better delineation between the layers of the choroid. EDI has confirmed the histopathological findings of choroidal thinning in myopes [1]; however, the interrelationship between choroidal layers and choroidal thinning has not been assessed.

Therefore, the aim of the present investigation is to analyse individual layers in relation to choroidal thinning of non-pathological myopic eyes, comparing the findings with age-matched healthy controls.

## Methods

### Study cohort

Retrospective analysis of EDI SD-OCT scans was performed for eyes with non-pathological myopia and compared to age-matched healthy subjects. Patients were examined between January 2013 and July 2015 at the L V Prasad Eye Institute, Hyderabad, India. Prior approval was obtained from the Institutional Review Board and informed consent was obtained from each subject for diagnostic and therapeutic procedures.

It has been shown that axial length has the most influence on choroidal thickness in eyes with high myopia [5]. For this reason, non-pathological myopia in this study was defined by an axial length greater than 25 mm and less then 27.5 mm without any clinical evidence of pathological changes at the posterior pole [6]. Eyes with an axial length above 27.5 mm or myopic eyes with any of the previous abnormalities or other myopia related macular pathologies such as vitreomacular traction, chorioretinal atrophy, or any history of choroidal neovascularization were excluded. Age matched emmetropic eyes identified by an axial length between 23.5 and 25 mm were recruited as a control group [6].

It is a protocol in our department to obtain OCT images for data mining purposes even from healthy patients. Axial length measurement was performed in each group using IOL Master (Carl Zeiss Meditec, Jena, Germany). Patients with systemic illnesses including diabetes, hypertension, or any other ocular or systemic disease were also excluded.

Collected data included demographics; details of the ocular and systemic exam were also recorded. Each subject’s BCVA was assessed using a Snellen chart, which was converted to logMAR (Logarithm of the Minimum Angle of Resolution) scale for statistical evaluation. The refractive error was measured for each subject using the Tonoref RKT-7000 autorefractometer (Nidek Inc, Aichi, Japan). Furthermore, the refractive error was measured by standard manual technique by an experienced optometrist.

### Image analysis

EDI SD-OCT imaging was acquired using Cirrus^®^ OCT Model 4000 (Carl Zeiss Meditec, Dublin, CA, USA). High quality images with signal strength of no less than 6 (on a 10-point scale) were included in the study. Choroidal thickness (CT) was defined as the distance between the hyperreflective line of the Bruch’s membrane and the innermost hyperreflective line of the chorio-scleral interface. CT measurements were obtained at: (a) fovea (SFCT), (b) 750 µm temporally to the fovea (T^750^CT), and (c) 750 µm nasally to the fovea (N^750^CT). Large choroid vessels were defined on cross-sectional OCT as those where the diameter exceeded 100 µm, as suggested by Branchini et al. [7].

**Fig. 1:**
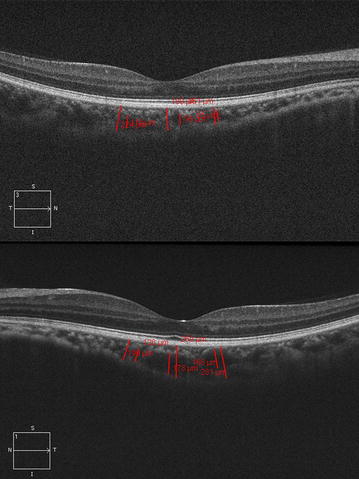
Enhanced depth imaging (EDI) optical coherence tomography (OCT) showing various choroidal measurements including choroidal thickness and large vessel thickness at subfovea, nasal 750 µm and temporal 750 µm in an eye with 20/20 vision and axial length of 27 mm

Large choroidal vessels (diameter more than 100 μm) located adjacent to the border of the chorio-scleral interface and within the vicinity of the CT measurement lines were identified. The same measurements were performed: perpendicular lines were traced from the innermost point of the large choroid vessel to the outer boundary of the vessel to measure the large choroidal vessel thickness at the fovea (SF-LCVT) and 750 µm on both sides of the fovea (N^750^LCVT and T^750^LCVT). Medium choroidal vessel thickness (MCVT) was considered as thickness of medium vessels and choriocapillaries, because the measurement of choriocapillaries separately is unreliable in cross-sectional SD-OCT with present imaging strategies. MCVT was calculated by subtracting large vessel thickness from choroidal thickness at all three locations (SF-MCVT, N^750^MCVT and T^750^MCVT). Due to some discrepancy in the resolution between horizontal and vertical scans, only horizontal (nasal to temporal) scans were analysed (Fig. 1). All measurements were performed by a single observer with an intra-observer repeatability of 0.98–0.99 for various measurements. The observer was blinded to the refractive error or the axial length of the patient while obtaining thickness measurements from OCT scans.

### Statistical analysis

Statistical analysis was performed using STATA version 11.0 software (Stata Corporation, College Station, TX, USA). Pearson correlation was performed to relate variables. Intraclass correlation was used to assess the intra-observer correlation of all the measurements of the choroidal vasculature. Variance was assessed using Levene’s test for equality of variances. Linear regression analysis was performed to determine whether age, axial length and sex were related to measures of choroidal thickness and choroidal vascular thickness. Generalized estimating equations (GEE) with robust standard errors were used to construct linear models to evaluate the relationship of age and axial lengths with covariates such as CT, LCVT and MCVT while adjusting for inter-eye correlation among individual patients. Significance was determined at p ≤ 0.05.

## Results

42 eyes of 31 patients with myopia: 16 males (mean age 48.3 ± 15 years) and 15 females (mean age 46.13 ± 15.63 years); and 57 eyes of 34 age-matched healthy eyes: 10 males (mean age 43.5 ± 36 years) and 24 females (mean age 42.38 ± 15.29 years) were included in this study (Tables 1, 2). Mean refractive error of each group for eyes with axial length of less than 25 mm and eyes with more than 25 mm was −2.75 ± 1.3 and 0.75 ± 0.65D respectively. Twelve patients had bilateral myopic eyes and twenty-three patients had bilaterally normal eyes. Fellow eyes of remaining subjects in both groups were excluded from the study due to poor image quality. All eyes in both groups had best corrected visual acuity (BCVA) of 20/20.

**Table 1:** Comparison of choroidal measurements between high myopic eyes and age-matched healthy controls

	Non-pathological myopic eyes	Age-matched healthy controls	p value
Age	46.13 ± 14.63	42.38 ± 15.29	0.34
Sex (females)	15	24	
Axial length	26.57 ± 1.27	23.59 ± 0.99	<0.001
Refractive error	−2.75 ± 1.3D	0.75 ± 0.65D	<0.001
SF-CT	353.8 ± 149.76	473.87 ± 142.68	<0.0001
N^750^ CT	314.95 ± 140.49	428.08 ± 153.50	<0.00013
T^750^ CT	358.65 ± 127.09	454.53 ± 142.12	<0.0003
SF-LCVT	202.59 ± 120.01	217.04 ± 76.70	0.232
N^750^ LCVT	190.99 ± 103.47	219.28 ± 78.83	0.061
T^750^ LCVT	212.17 ± 86.59	211.85 ± 73.48	0.492
SF-MCVT	151.201 ± 70.02	256.82 ± 92.50	<0.0001
N^750^ MCVT	123.95 ± 66.32	208.8 ± 106.84	<0.0001
T^750^ MCVT	146.48 ± 71.20	242.68 ± 102.32	<0.001

*SF CT* subfoveal choroidal thickness, *SF LCVT* subfoveal large vessel choroidal thickness, *N* ^*750*^ *CT* choroidal thickness 750 µm from macula, *N* ^*750*^ *LCVT* nasal large vessel choroidal thickness 750 µm from macula, *T* ^*750*^ *CT* temporal choroidal thickness 750 µm from macula, *T* ^*750*^ *LVT* temporal large vessel choroidal thickness 750 µm from macula, *SF LCVT* subfoveal medium vessel choroidal thickness, *N* ^*750*^ *MCVT* nasal medium vessel choroidal thickness 750 µm from macula, *T* ^*750*^ *MCVT* temporal medium vessel choroidal thickness 750 µm from macula

**Table 2:** Relationship of age and axial length with choroidal thickness measurements

	Age			Axial length		
	Coefficient	p value	r^2^	Coefficient	p value	r^2^
SF-CT	−3.7	0.0003	0.12	−38.2	<0.0001	0.2
N^750^ CT	−2.81	0.0068	0.07	−35.5	<0.0001	0.17
T^750^ CT	−3.73	0.0001	0.15	−32.9	<0.0001	0.18
SF-LCVT	−2.26	0.0003	0.12	−8.4	0.19	0.02
N^750^ LCVT	−1.22	0.04	0.04	−10	0.04	0.04
T^750^ LCVT	−1.21	0.02	0.05	−5.4	0.2	0.01
SF-MCVT	−1.43	0.028	0.04	−29.79	<0.0001	0.31
N^750^ MCVT	−1.58	0.017	0.056	−25.5	<0.0001	0.21
T^750^ MCVT	−2.51	0.0001	0.13	−27.4	<0.0001	0.24

*SF CT* subfoveal choroidal thickness, *SF LCVT* subfoveal large vessel choroidal thickness, *N* ^*750*^ *CT* choroidal thickness 750 µm from macula, *N* ^*750*^ *LCVT* nasal large vessel choroidal thickness 750 µm from macula, *T* ^*750*^ *CT* temporal choroidal thickness 750 µm from macula, *T* ^*750*^ *LVT* temporal large vessel choroidal thickness 750 µm from macula, *SF LCVT* subfoveal medium vessel choroidal thickness, *N* ^*750*^ *MCVT* nasal medium vessel choroidal thickness 750 µm from macula, *T* ^*750*^ *MCVT* temporal medium vessel choroidal thickness 750 µm from macula

### Comparison between high myopes and age-matched controls

In patients with non-pathological myopia, CT at all locations was significantly thinner than in patients with normal eyes. Specifically, the CT 750 µm (N^750^CT) nasal to the fovea was the thinnest region in myopes; this measured 314.95 versus 428.08 µm in age-matched controls (p < 0.0001). The SFCT was 353.8 ± 149.8 versus 473.9 ± 142.7 µm (p < 0.0001), while the CT 750 µm temporal to the fovea T^750^CT was the thickest point of the choroid in myopes, but still thinner compared to normal eyes (358.6 ± 127.1 vs. 454.5 ± 142.1 µm, p = 0.0003, Table 1).

However, LCVT did not vary between patients with myopic eyes and patients with normal eyes at all locations. Measurements between patients with myopia and those with normal eyes, respectively, were the following: SF-LCVT 202.6 ± 120.0 versus 217.0 ± 76.7 µm (p = 0.232); N^750^LCVT 190.9 ± 103.5 versus 219.3 ± 78.8 µm (p = 0.61) and T^750^LCVT 212.17 ± 86.6 versus 211.8 ± 73.5 µm (p = 0.492) (Table 1).

Compared to normal eyes, myopic eyes showed significant thinning of the MCVT at all locations (p < 0.0001). Measurements between patients with myopia and those with normal eyes, respectively, were the following: SF-MCVT 151.2 ± 70.0 versus 256.8 ± 92.5 µm (p < 0.001), N^750^MCVT 123.9 ± 66.3 versus 208.8 ± 106.8 µm (p < 0.001) and T^750^MCVT 146.48 ± 71.2 versus 242.6 ± 102.3 µm (p < 0.001) (Table 1).

### Relationship between axial length and choroidal thickness measurements (Fig. 2)

Mean axial length in myopic eyes was greater than in normal eyes (26.6 ± 1.3 vs. 23.6 ± 1.0 µm, p < 0.001). Axial length measured in patients with myopic eyes was related to gender (male: 27.0 ± 1.4 mm vs. female: 26.1 ± 0.9 mm, p = 0.026). Linear regression showed that every mm of increase in axial length caused 38.2, 35.5 and 32.9 µm thinning at the subfovea, 750 µm nasally and 750 µm temporally to the fovea, respectively (p < 0.0001 at all locations).

**Fig. 2:**
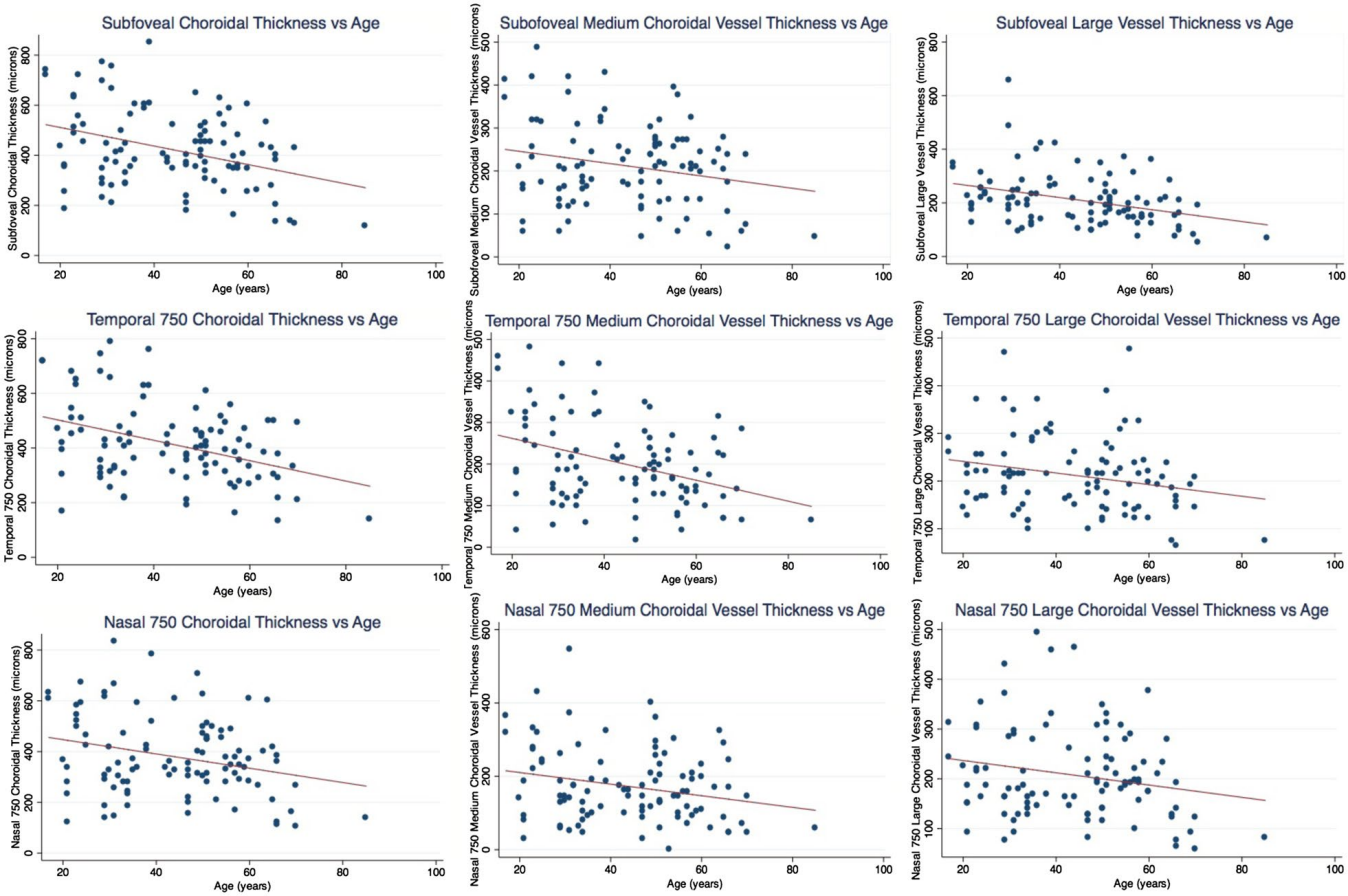
Graphs showing relationship of various choroidal measurements with axial length

The most significant thinning was noted in MCVT with every mm increase in axial length: approximately 30 µm at all locations (29.79, 25.5 and 27.4 µm at the subfovea, 750 µm nasally and 750 µm temporally to the fovea, respectively, p < 0.0001 at all locations). However, LCVT was not significantly correlated to the axial length. Every mm of axial length caused 8.4 µm, 10 µm and 5.4 µm thinning of LCVT at the subfovea (p = 0.19), 750 µm nasally (p = 0.04) and 750 µm temporally (p = 0.2) to the fovea, respectively (p < 0.05 at all locations) (Table 2).

### Relationship between age and choroidal thickness measurements (Fig. 3)

Linear regression analysis showed that with every 10 years of increase in age, SF-CT, N^750^CT and T^750^CT decreased by 37 µm (p = 0.0003), 28.1 µm (p = 0.0068) and 37.3 µm (p = 0.0001), respectively.

**Fig. 3:**
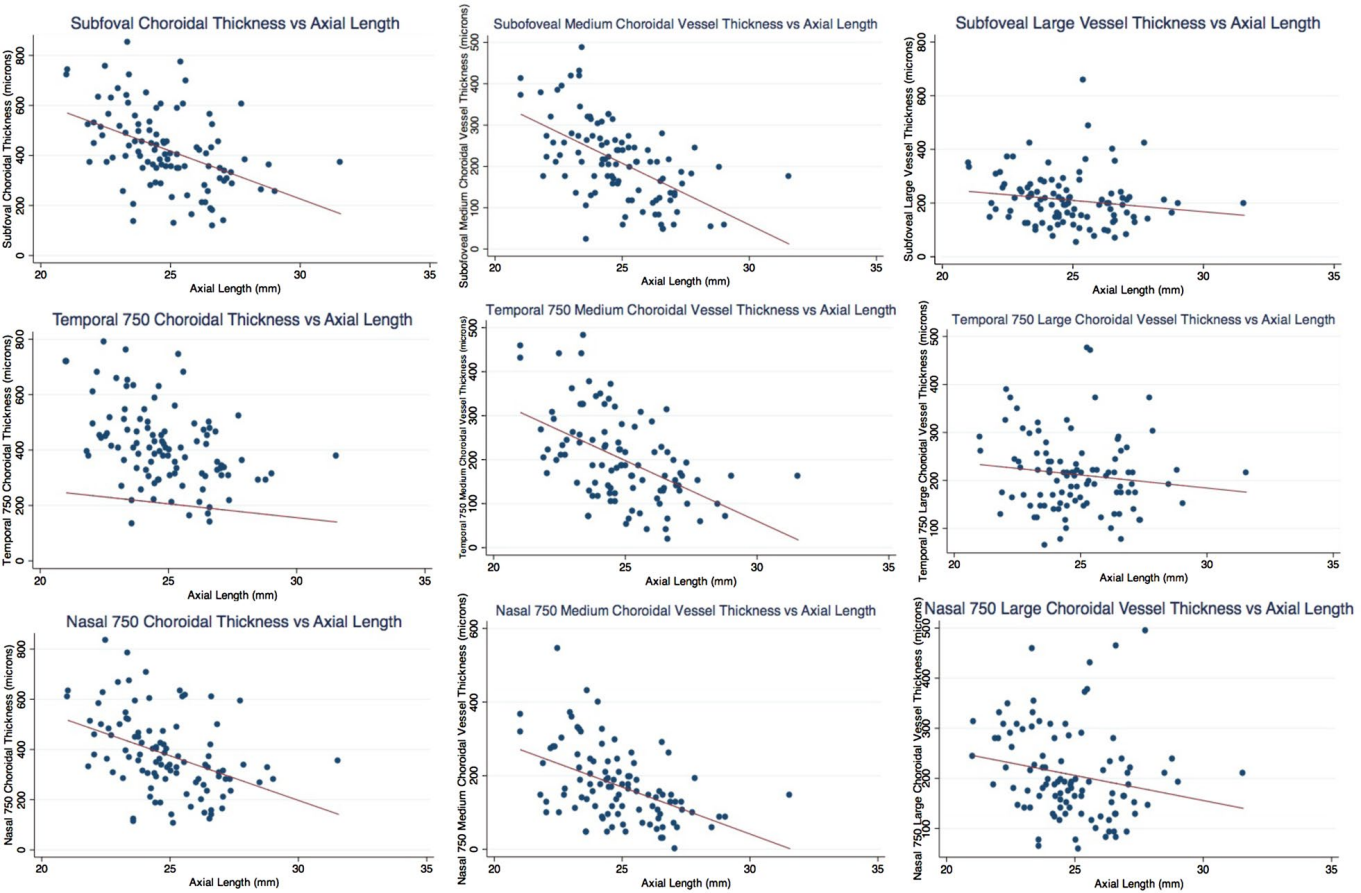
Graphs showing relationship of various choroidal measurements with age

In regards to individual layer thicknesses, both layers had significant correlation with age. Every 10-year increment in age resulted in a decline of LCVT by 22.6 µm (p = 0.0003), 12.2 µm (p = 0.04) and 12.1 µm (p = 0.02), when measured at the subfovea, 750 µm nasally and 750 µm temporally to the fovea, respectively. In regards to MCVT, every 10-year increment in age resulted in a decline of MCVT by 14.3 µm (p = 0.028), 15.8 µm (p = 0.017) and 25.1 µm (p = 0.0001), when measured at the subfovea, 750 µm nasally and 750 µm temporally to the fovea, respectively (Table 2).

### Relationship between gender and choroidal thickness measurements

In myopic eyes, gender was not related to choroidal thickness measurements in the subfoveal or temporal aspects, as follows: SF-CT male 390.6 ± 152.2 versus female 311.5 ± 152.2 µm (p = 0.084), and T^750^CT male 387.6 ± 133.3 versus female 325.4 ± 133.3 µm (p = 0.110). However, myopic eyes in males tended to have greater choroidal thickness than in females when measured nasally to the fovea N^750^CT male 355.1 ± 143.5 µm versus female 268.8 ± 125.0 µm (p = 0.04).

Gender did not seem to be related to LCVT because the differences were not statistically significant. SF-LCVT male 235.3 ± 139.0 versus female 165.0 ± 139.0 µm (p = 0.054), N^750^LCVT male 218.9 ± 115.2 versus female 158.9 ± 115.2 µm (p = 0.057), and T^750^LCVT male 227.5 ± 89.6 versus female 194.6 ± 89.6 µm (p = 0.218).

Similarly, gender was not related to LCVT, as follows: SF-MCVT male 155.3 ± 64.3 versus female 146.5 ± 77.5 µm (p = 0.683), N^750^MCVT male 136.2 ± 72.3 versus female 109.9 ± 57.3 µm (p = 0.2), and T^750^MCVT male 160.1 ± 71.9 versus female 130.8 ± 68.8 µm (p = 0.181).

## Discussion

Similar to previous reports [7, 8], using EDI SD-OCT in patients with non-pathological myopia and age-matched normal subjects, we also found that total choroidal thickness is smaller in eyes with myopia. Furthermore, our findings demonstrate that large vessel choroidal thickness does not differ between patients with myopia and those with normal eyes, whilst medium layer choroidal thickness is significantly reduced in myopic eyes. Finally, this study also demonstrates that in patients with myopia, medium vessel layers thickness seems to have consistent and stronger correlation with age and axial length.

Histological studies have also demonstrated that myopic eyes have thin choroid [4]. Whilst the mechanism and pathobiology of choroidal thinning has not been established, it has been postulated that choroidal thinning is associated with perturbation in vasculature. Histological studies of myopia describe a reduction in large choroidal vessels [4]. Likewise, indocyanine green angiography studies also report a reduction and attenuation in large choroidal vessels with myopia [9, 10]. A number of recent small-scale investigations have hinted at reduced vascular thickness in the choroidal related to changes in myopia. Using a chick model, Shih et al. [10] demonstrated that induced myopia led to increased axial length, as is also seen in humans. Chick studies further report that choroid thickness is influenced by circulatory dynamics [11], and that myopia results in lower blood vessel density and smaller vessel diameter [12, 13].

Based upon these studies, it has been suggested that either (a) large vasculature atrophy may occur secondary to axial elongation and axial stretching [14], which in turn alters the perfusion of the choroid; or (b) atrophy of large vasculature causes choroidal thinning [8]. Seemingly contrary to these hypotheses and findings, data from our cohort demonstrated that choroidal thinning was related, not to large vessel choroidal thinning, but rather to medium vessel choroidal thinning. To the best of our knowledge, this is the first investigation to demonstrate a relationship between total choroidal thickness and medium vessel choroidal thickness in myopia in vivo.

It is important to further understand the relationship between choroidal thinning, choroidal vascularization and ocular blood flow, in order to develop treatment approaches to chorioretinal atrophy and choroidal neovascularization (CNV) in pathological myopia. Recent reports have suggested that chorioretinal atrophy, possibly related to hypoxia, is associated with choroidal thinning, causing degenerative high myopia, which is a characteristic of pathological myopia and thus a major threat to vision [1, 15]. It is suggested that atrophy of large vasculature may cause hypoxic conditions that promote CNV and choroidal thinning. In fact, El Matri et al. [16] analysed the interrelationship between subfoveal choroidal thickness and history of CNV in high myopic eyes. Findings showed that in high myopic eyes, those with CNV had markedly thinner choroid than eyes without CNV. The authors postulated that CNV in eyes with high myopia might be a hypoxic response to loss of vascularization [16]. Current treatment for CNV emphasizes blocking vascular endothelial growth factor (VEGF) signalling [17], which has been associated with post-treatment choroidal thinning [18].

Although EDI SD-OCT imaging of the choroid is already a well-established area of investigation, the role of medium vascular choroidal layer (also known as Sattler’s layer) has been so far overlooked. However, many of the changes associated with choroidal thinning and large vessel choroidal atrophy have been related to age [19]. Progressive age-related macular degeneration thought to be caused by atherosclerosis may result in the development of CNV [20], and atherosclerosis is more likely to occur in vessels with low shear stress [21, 22]. Within choroidal vasculature, shear stress is postulated to decrease from the large vessels to the medium and further to the choriocapillaris, much like coronary cardiac circulation [23, 24]. Therefore, the medium vessel choroidal layer and choriocapillaris may develop arteriosclerosis prior to large vessel choroidal layer involvement. Choroidal blood flow is significantly reduced in high myopic eyes, and is thought to be related to vascular resistance [25]. Arteriosclerosis of the medium vessel choroidal layer may lead to hypoxia and thinning of the choroid.

Additionally, a recent study [25] evaluated choroidal blood flow alteration in human eyes with high myopia by analysing the pulsatile components of ocular blood flow and found that high myopia correlates with changes in pulsatile ocular blood flow, pulse amplitude, and pulse volume. The authors hypothesized that narrowing of the choroidal vessel diameter and increased rigidity of the choroidal vessel wall cause blood flow changes. They further hypothesized that changes in axial length and the possible influence of these changes on the physical properties of choroidal vessels increases risk for ocular vascular diseases in high myopia [25]. Importantly, blood flow and total vascular area are two distinct parameters, and alterations in one do not require changes in the other. It was observed that axial length of myopic eyes in the current study cohort is slightly less than that reported by other studies where a reduction in large vasculature was noted [8].

The primary limitations of this study revolve around its retrospective nature and relatively small number of patients. In addition, we only included patients with myopia and no secondary complications. Therefore, our results cannot be applicable to eyes with changes related to pathological myopia or with any complications secondary to high myopia. Choriocapillaries play a major role in the pathology of high myopia; however, choriocapillaries cannot be differentiated from medium vessel choroidal layer using present imaging strategies on SD-OCT, therefore, we assessed choriocapillaries and medium vessel choroidal layer as one single measurement. Another limitation was that we did not account for the impact of magnification factors on the results we analyzed. While measurements were collected at 750 microns from the fovea the true distance of these measurements from the fovea might vary depending on the axial length of the subjects. Furthermore, current reports demonstrate that there is a diurnal pattern of change in choroidal thickness; however, the exact pattern remains undetermined [26–28]. We were not able to obtain images at fixed intervals to determine the effect of diurnal variation on vascular or stromal thickness. Finally, automated choroidal thickness analysis of individual choroidal layers is not commercially available yet, for this, manual calculations are required. Of note, manual measurements might be associated with errors in measurements due to inaccurate definition of choroid-scleral boundaries.”

In non-pathologic myopic eyes, using EDI SD-OCT, we report evidence of medium vessel choroidal layer thinning in the absence of large vessel choroidal layer thinning. Further studies are needed to investigate changes in individual choroidal layers in a longitudinal study, as well as studies involving eyes with pathologic myopia and secondary complications of myopia.
